# Anlotinib plus platinum‐etoposide as a first‐line treatment for extensive‐stage small cell lung cancer: A single‐arm trial

**DOI:** 10.1002/cam4.4736

**Published:** 2022-05-08

**Authors:** Pengbo Deng, Chengping Hu, Cen Chen, Liming Cao, Qihua Gu, Jian An, Ling Qin, Min Li, Baimei He, Juan Jiang, Huaping Yang

**Affiliations:** ^1^ Department of Respiratory Medicine National Key Clinical Specialty, Branch of National Clinical Research Center for Respiratory Disease, Xiangya Hospital, Central South University Changsha China; ^2^ Xiangya Lung Cancer Center Xiangya Hospital, Central South University Changsha China; ^3^ Center of Respiratory Medicine Xiangya Hospital, Central South University Changsha China; ^4^ Clinical Research Center for Respiratory Diseases in Hunan Province Changsha China; ^5^ Hunan Engineering Research Center for Intelligent Diagnosis and Treatment of Respiratory Disease Changsha China; ^6^ National Clinical Research Center for Geriatric Disorders Xiangya Hospital, Central South University Changsha China; ^7^ Department of Respiratory Medicine the First People's Hospital of Changde City Changde China; ^8^ Department of Geriatric Medicine Xiangya Hospital, Central South University Changsha China

**Keywords:** anlotinib, extensive stage, first‐line, phase II trial, SCLC

## Abstract

**Background:**

Anlotinib as a third‐line or beyond therapy for extensive‐stage small‐cell lung cancer (ES‐SCLC) was studied. This single‐arm phase II trial was to investigate the value of anlotinib plus platinum‐etoposide as first‐line treatment in ES SCLC.

**Methods:**

The primary endpoint was progression‐free survival (PFS) and objective response rate (ORR). The secondary endpoints included overall survival (OS), disease control rate (DCR), time to progression (TTP), duration of remission (DoR), and safety. The subgroups of preset liver metastasis and brain metastasis were analyzed.

**Results:**

In 35 ES‐SCLC patients, the median PFS, ORR, DCR, and OS were 8.02 months [95% confidence interval (CI): 6.90–9.66], 85.71% (95% CI: 69.74–95.19), 94.29% (95% CI: 80.84–99.30), and 15.87 months (95% CI: 10.38–18.89), respectively. The median PFS in the liver metastasis and brain metastasis subgroups was 7.33 months (95% CI: 4.76–9.69) and 7.34 months (95% CI: 5.68–9.20), respectively. The most common AEs with grade 3–4 were hand–foot syndrome (17%), granulocytosis (17%), stomatitis (14%), hypertriglyceridemia (11%), hypercholesterolemia (11%), as well as nausea and vomiting (11%), and no grade 5 AEs were recorded.

**Conclusions:**

Anlotinib combined with platinum‐etoposide provided an effective and safe therapy for patients with ES‐SCLC.

## Graphical abstract

Abstract graphic for the table of contents.

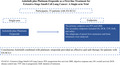

## 1 INTRODUCTION

Lung cancer is the leading cause of death worldwide, and small cell lung cancer (SCLC) accounts for approximately 15% of all lung cancers.^1^ The majority of patients with SCLC present with extensive‐stage disease, and their prognosis remains poor.^2^ The 5‐year survival rate of patients in the extensive stage is <5%, associating with a worse prognosis.^3^ The median overall survival (OS) of patients with extensive‐stage SCLC (ES‐SCLC) who are treated with standard frontline chemotherapy is within 10 months.^4^, ^5^

For the majority of patients with ES‐SCLC, the first‐line treatment includes immune checkpoint inhibitors (ICIs) plus etoposide and cisplatin (EP), and the mentioned treatment has been confirmed by the 2021 guidelines published by the National Comprehensive Cancer Network and the Chinese Society of Clinical Oncology. However, patients with both high tumor mutational burden (TMB) and a programmed death‐ligand 1 (PD‐L1) expression ≥50% tumor cells had a higher response rate (75%).^6^ when treated with ICI + EP than patients with only one of these factors. The relapse mainly occurred within 6 months after completing the initial treatment, and the median OS was about 10 months.^7^ A meta‐analysis demonstrated that the median progression‐free survival/overall survival (PFS/OS) was 5.5/9.6 and 5.3/9.4 months, and the objective response rate (ORR) was 67.1% and 66.0% for EP and EC in the first‐line treatment of SCLC, respectively.^8^ Similar results were also reported by Japanese scholars, in which the median PFS/OS was 5.2/10.6 months (EC) and 4.7/9.9 months (EP), and the ORR was 73.0%.^9^

Although the results of immunotherapy are promising, the CASPIAN trial assessed the durvalumab plus platinum‐etoposide regimen and showed that OS was significantly longer than that in the platinum‐etoposide group (13.0 vs. 10.3 months).^10^ The IMpower‐133 trial revealed that the median PFS/OS was 5.2/12.3 months in the atezolizumab plus platinum‐etoposide group and 4.3/10.3 months in the placebo group.^6^ Even though ICI + chemotherapy had exhibited a noticeably longer OS compared to that in the conventional chemotherapy, its efficacy significantly varies for SCLC patients receiving immunotherapy (i.e., some patients may have a long OS), and the existing predictors of therapeutic efficacy are not highly robust, indicating the necessity of exploration of further reliable biomarkers. Furthermore, the limitations of the administration of ICIs in the first‐line treatment of patients with ES‐SCLC could not be neglected. The lower expression level of PD‐L1 and the lack of an association of the mentioned expression level with therapeutic benefit indicated that the expression level of PD‐L1 was unlikely to be a predictor of therapeutic efficacy.

Administration of anti‐vascular‐targeted therapies combined with chemotherapy has been reported in the first‐line treatment of SCLC patients. The pooled survival data from the previous first‐line trials revealed a median PFS time of 6–7 months and a median OS time of ~11 months for combined treatment of bevacizumab and chemotherapy, which were similar to those results reported in other trials concentrated on chemotherapy only.^11^ This result was similar to that obtained from administration of apatinib plus chemotherapy, with a PFS of 6.0 months and an OS of 12 months in a randomized phase 2 trial for patients with ES‐SCLC.^12^

Anlotinib is an oral tyrosine kinase inhibitor targeting c‐kit, platelet‐derived growth factor receptors, fibroblast growth factor receptor, and vascular endothelial growth factor receptor.^13^ On May 8, 2018, the China National Medical Products Administration (NMPA) approved anlotinib, an orally administered antiangiogenesis inhibitor, for the treatment of patients with advanced non‐small cell lung cancer (NSCLC) who have progressed after treatment with two or more lines of prior systemic chemotherapy.^14^ Anlotinib is currently undergoing careful exploration as a treatment option for SCLC, soft tissue sarcoma, colorectal cancer, etc. The results of phase II clinical trial (ALTER 1202) on administration of anlotinib as a third‐line therapy for SCLC patients were presented.^15^ A placebo‐controlled, multicenter study showed a significant improvement in the PFS and OS of patients who received anlotinib treatment.

This clinical trial aimed to investigate the efficacy and safety of anlotinib plus platinum‐etoposide, as the first‐line treatment, for patients with ES‐SCLC.

## 2 MATERIAL AND METHODS

### 2.1 Patients and study design

This single‐arm phase II trial enrolled patients with ES‐SCLC, without prior systematic chemotherapy or ICI therapy, who were admitted to Xiangya Hospital Affiliated to the Central South University, from August 23, 2018, to January 16, 2020. The study was conducted in accordance with the International Conference on Harmonization Good Clinical Practice Guideline, the Declaration of Helsinki, and applicable local regulations with approval from an independent ethics committee or institutional review boards. The protocol and all modifications were approved by the Medical Ethics Committee of Xiangya Hospital, Central South University (ClinicalTrials. Gov number, NCT04675697). All patients provided written informed consent for participation.

The inclusion criteria were as follows: (1) patients who aged 18–70 years old; (2) patients who were histologically confirmed with ES‐SCLC according to the diagnostic criteria presented by the Veterans Administration Lung Study Group and the Response Evaluation Criteria in Solid Tumors (RECIST, ver. 1.1); (3) patients with an Eastern Cooperative Oncology Group (ECOG) performance‐status (PS) score of 0 or 1, and 4) patients who had not received previous systemic treatment for EC‐SCLC and had an expected survival time ≥3 months. Patients with asymptomatic central nervous system (CNS) metastases who underwent therapy were also eligible under some circumstances (see the Appendix S1). The exclusion criteria were having a history of mixed small cell carcinoma and NSCLC with active CNS metastasis and/or cancerous meningitis during screening. The inclusion and exclusion criteria are summarized in the Appendix S1.

### 2.2 Interventions

The platinum‐etoposide regimen consisted of administration of etoposide 100 mg/m,^2^ days 1–3 of the 21‐day cycle, with investigators' choice of either cisplatin (75–80 mg/m^2^, Q3W) or carboplatin (AUC = 5–6, Q3W), and anlotinib treatment of 12‐mg qd from day 1 to day 14 of a 21‐day cycle. Eligible patients received anlotinib plus platinum‐etoposide for 4–6 cycles, followed by maintenance therapy with anlotinib.

Discontinuity/Withdrawal: All patients who signed the informed consent had the right to withdraw from the study at any time. Any patient who withdrew from the study for any reason at any time and without completing all observations of the clinical study was deemed as a case of loss. The reasons for loss and withdrawal of patients included the following: (1) adverse events (AEs); (2) serious breach of protocol; (3) loss of follow‐up; (4) voluntary withdrawal of informed consent by patients; (5) situations in which, researchers believed, the patient should withdraw from the study; and (6) others (e.g., pregnancy).

Efficacy evaluation was conducted every 6 weeks during the post‐enrolment enhancement period (anlotinib +EP/EC) after enrolment and every 9 weeks (anlotinib maintenance) following in the maintenance period. The tumor evaluation schedule was calculated on a calendar basis from random days, regardless of whether the treatment was delayed or not. For other parts (including the brain and bone) known or suspected to have lesions, patients underwent imaging examination when clinical indications were present. The evaluation was also performed when the disease was suspected to have progressed (e.g., deteriorated symptoms) or when the patient discontinues the treatment (if tumor evaluation was not performed in the previous 4 weeks at most). For patients suspected to have disease progression before the next scheduled tumor evaluation, an extra tumor evaluation was performed. AEs were graded according to the National Cancer Institute Common Terminology Criteria (version 4.02).

### 2.3 Outcomes

The primary endpoint was the PFS and ORR [complete response (CR) + partial response (PR)], and the secondary endpoints were OS, the disease control rate [DCR; CR + PR + stable disease (SD)], including the time to progression (TTP), duration of remission (DoR), and safety of the whole therapeutic process of anlotinib plus EP as a first‐line therapy for patients with EC‐SCLC. In the liver metastasis and brain metastasis subgroups, the OS, PFS, ORR, and DCR were analyzed. PFS, ORR, and DoR were assessed using the RECIST (ver. 1.1), and the ORR was confirmed twice according to the RECIST (ver. 1.1).

### 2.4 Statistical analysis

Efficacy‐based data were analyzed on an intention‐to‐treat basis, regardless of whether patients have received treatment or not. All patients who received at least one dose of the therapy were included in the safety analysis. Periodic safety monitoring and interim efficacy assessment were conducted by an independent data monitoring committee.

ORR and PFS do not share the α value but historical controls were given from the previously study.^16^ Hence, the maximum value was calculated separately. The iPASS15 software was used, two‐sided, ORR was preset to 80% compared with 55% in control, β = 0.05, α = 0.2, and the sample size was 33. The preset inclusion time was 18 months, PFS was 9 months compared with 5.2 months in control, β = 0.05, α = 0.2, and the sample size was 35 cases (with 27 events). Therefore, the final sample size of the trial was determined to be 35 (excluding the shedding rate).

Statistical analysis was performed using the SAS 9.4 software (SAS Institute, NC, USA). Continuous variables were expressed as mean ± standard deviation (SD), and categorical variables were presented as percentage. PFS and ORR were analyzed using the log‐rank test with hazard ratios (HRs) and 95% confidence intervals (95% CIs), which were estimated using the Cox proportional hazards model. The Kaplan–Meier method was also used to estimate OS, TTP, and DoR. A two‐tailed *P*‐value <0.05 was considered statistically significant.

## 3 RESULTS

### 3.1 Patients' demographic and clinical characteristics

**FIGURE 1:**
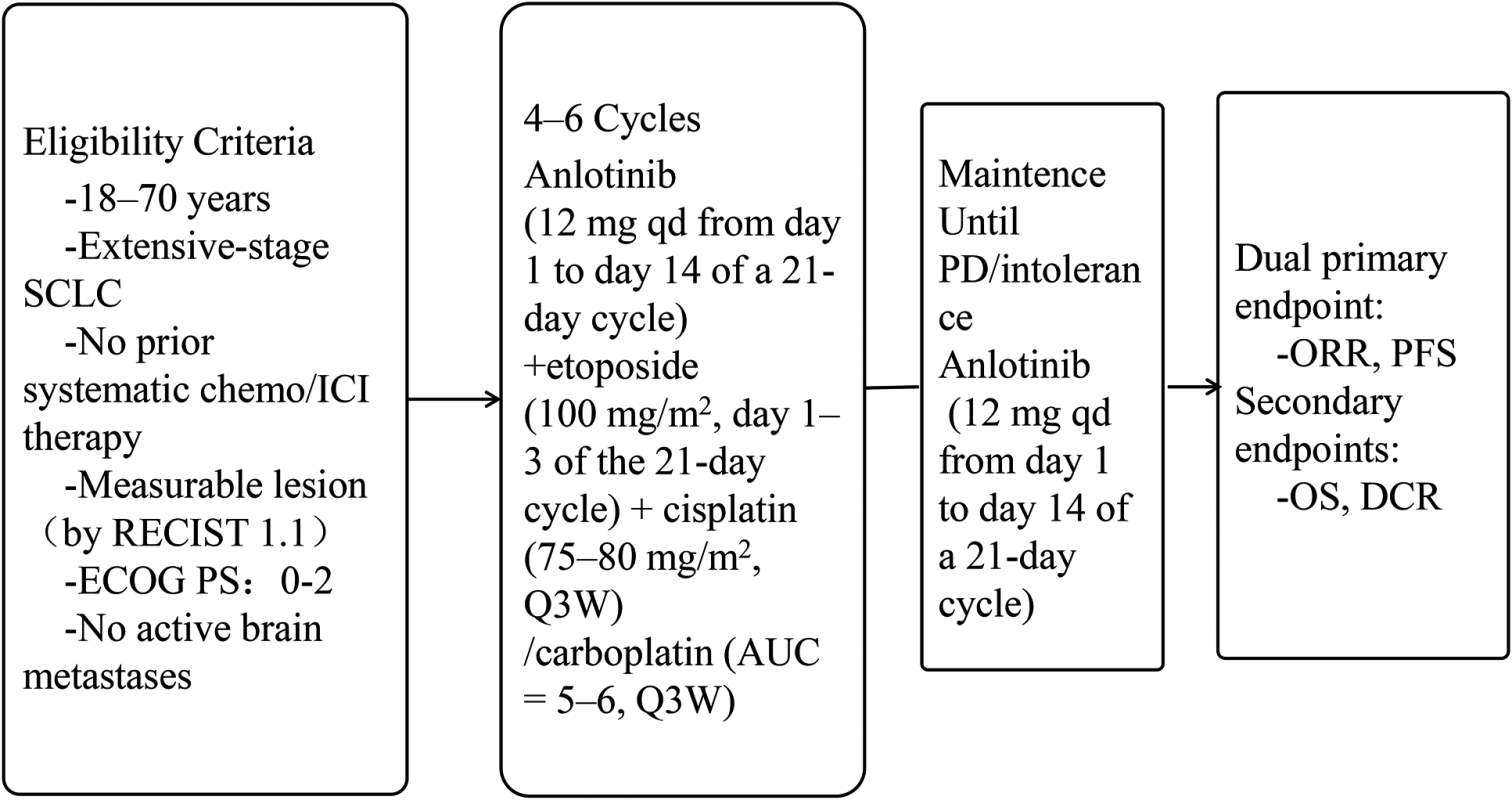
Flow chart of the single‐arm phase II trial

Of the 35 patients with extensive‐stage SCLC enrolled between August 23, 2018, and January 16, 2020, 36 patients were enrolled and 35 patients were available for efficacy analysis (n = 35, demographics in Table 1). The data cutoff was: December 2, 2020. The median age was 59 years. The male–female ratio was very different, at 94.29% versus 5.71%. The majority of patients (88.57%) had a good PS score of 0–1. Besides, seven patients received prophylactic cranial irradiation, and three cases received pulmonary irradiation (Table 1). The flow chart of the single‐arm phase II trial is shown in Figure 1.

**TABLE 1:** Baseline patient demographics and disease characteristics

Variables	Anlotinib plus platinum‐etoposide (*n* = 35)
Median age (year)	59 (44–75)
Age group (year)	
<60	18 (51.43%)
≥60	17 (48.57%)
Sex	
Men	33 (94.29%)
Women	2 (5.71%)
Disease stage	
III	1 (2.86%)
IV	34 (97.14%)
ECOG PS	
0	31 (88.57%)
1	4 (11.43%)
Brain or CNS metastases	
Yes	7 (20.00%)
No	28 (80.00%)
Liver metastases	
Yes	13 (37.14%)
No	22 (63.86%)
Smoking history	
Never smoker	7 (20.00%)
Former smoker	12 (34.29%)
Current smoker	16 (45.71%)
Prior radiotherapy treatment	
Prophylactic cranial irradiation	7 (20%)
Therapeutic cranial irradiation	5 (24%)
Therapeutic pulmonary irradiation	3 (8.6%)

### 3.2 Efficacy

**FIGURE 2:**
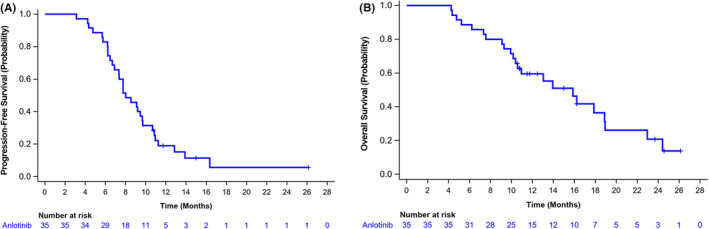
Progression‐free survival and overall survival of Kaplan–Meier curves in the whole patient cohort. (A) Median PFS was 8.02 months (95% CI: 6.90–9.66). (B) Median OS was 15.87 months (95% CI: 10.38–18.89) in the whole patient cohort

The median PFS was 8.02 months (95% CI: 6.90 m, 9.66 m) and the median OS was 15.87 months (95% CI: 10.38 m, 18.89 m) in the whole patient cohort (Figure 2) with an event rate of 65.71%. In addition, ORR and DCR were 85.71% (95% CI: 69.74–95.19) and 94.29% (95% CI: 80.84–99.30), respectively. With consideration of liver lesions as target lesions, ORR and DCR were 76.92% (95% CI: 46.19–94.96) and 84.62% (95%CI: 54.55–98.08), respectively. A separate evaluation of the brain's lesions showed that the patients with brain metastasis were 87.50% (95% CI: 47.35, 99.68) and 100.00% (95% CI: 63.06, 100.00) (Table 2).

**TABLE 2:** Response rate

	ITT (*n* = 35)	Liver metastases group (*n* = 13)	Brain metastases group (*n* = 8)
CR, *n* (%)	1 (2.86)	0	0
PR, *n* (%)	29 (82.86)	10 (76.92)	7 (87.50)
SD, *n* (%)	3 (8.57)	1 (7.69)	1 (12.50)
PD, *n* (%)	2 (5.71)	2 (15.38)	0
ORR (95% CI)	85.71 (69.74, 95.19)	76.92 (46.19, 94.96)	87.50 (47.35, 99.68)
DCR (95% CI)	94.29 (80.84, 99.30)	84.62 (54.55, 98.08)	100.00 (63.06, 100.00)

Abbreviations: DCR, Disease control rate; NE, not evaluable; ORR, objective response rate; PD, progressive disease; PR, partial response; SD, stable disease.

**FIGURE 3:**
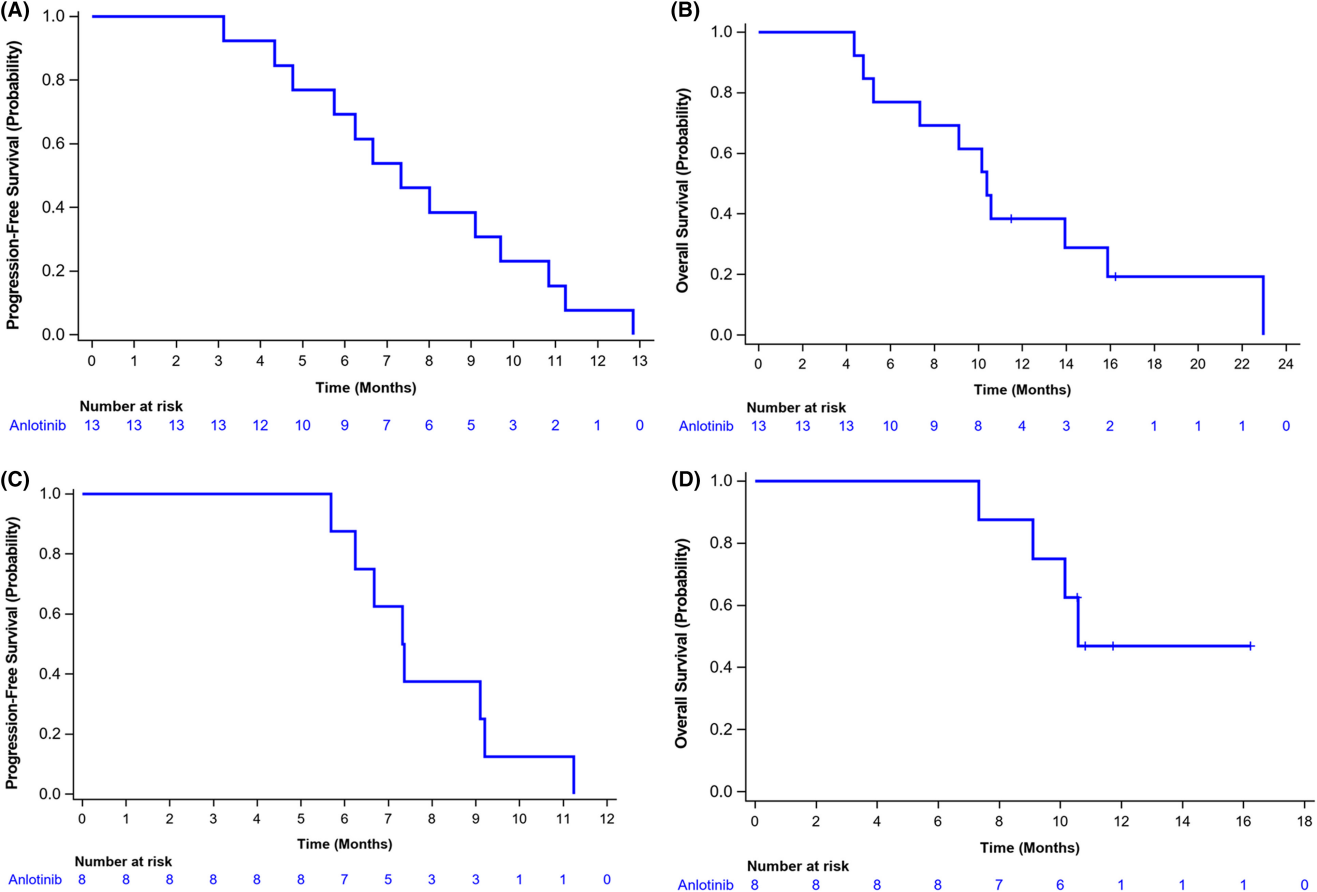
Progression‐free survival (PFS) of Kaplan–Meier curves in patients with hepatic metastases and brain metastases. (A) Median of PFS in patients with liver metastasis was 7.33 months (95% CI: 4.76–9.69). (B) Median of OS was 10.38 months (95% Cl: 5.22–15.87). (C) Median of PFS in patients with brain metastases was 7.34 months (95% CI: 5.68–9.20). (D) Median of OS was 10.58 months (95% Cl: 7.33, NE)

As for the clinical benefit, the median PFS in the liver metastasis subgroup was 7.33 months (95% CI: 4.76 m, 9.69 m), and the median PFS in the brain metastasis subgroup was 7.34 months (95% CI: 5.68 m, 9.20 m) (Figure 3). Moreover, the OS was 10.38 months (95% CI: 5.22 m‐15.87 m) and 10.58 months (95% CI: 7.33 m, NE) in the liver metastasis and brain metastasis subgroups, respectively.

### 3.3 Safety

The incidence of adverse events (AEs) was 97% in the whole cohort. In all patients, 40% had 3–4 grade AEs and 11% had serious AEs (SAEs). The most common AEs with grade 3–4 were hand–foot syndrome (17%), granulocytosis (17%), stomatitis (14%), hypertriglyceridemia (11%), hypercholesterolemia (11%), as well as nausea and vomiting (11%) (Table 3). Anlotinib dose was also adjusted/discontinued in 15 subjects (9 to 10 mg, 5 to 8 mg, and 1 discontinuation). Importantly, no grade 5 AEs were recorded.

**TABLE 3:** Treatment‐related adverse events (AE) of any cause

Variable	Any grade, No. (%)	3–4 Grade, No. (%)	Serious AE, No. (%)
Hypertriglyceridemia	19 (54)	4 (11)	0 (0)
Stomatitis	12 (34)	5 (14)	0 (0)
Hypercholesterolemia	11 (31)	0 (0)	0 (0)
Hand–foot syndrome	10 (29)	6 (17)	0 (0)
Granulocytosis	10 (29)	6 (17)	0 (0)
leukopenia	7 (20)	3 (9)	1 (3)
Hemoptysis	7 (20)	0 (0)	0 (0)
Fatigue	7 (20)	0 (0)	0 (0)
Nausea and vomiting	5 (14)	4 (11)	1 (3)
Hypertension	5 (14)	3 (9)	0 (0)
Diarrhea	5 (14)	0 (0)	0 (0)
Anemia	5 (14)	0 (0)	1 (3)
Loss of appetite	4 (11)	2 (6)	0 (0)
Proteinuria	3 (9)	0 (0)	0 (0)
Rash	2 (6)	0 (0)	0 (0)
Scalp rash	2 (6)	0 (0)	0 (0)
Thirst	1 (3)	1 (3)	0 (0)
Anasarca	1 (3)	1 (3)	0 (0)
Liquid pneumothorax	1 (3)	1 (3)	1 (3)
Total AEs	34 (97)	14 (40)	4 (11)

*Note*: The date of data cutoff was Dec 2, 2020. Multiple occurrences of the same adverse event in one patient were counted once at the highest grade for the preferred term. The incidence of treatment‐related adverse events associated with any component of the trial regimen is shown.

Abbreviation: AEs: adverse events.

## 4 DISCUSSION

This exploratory study was the first to prospectively assessed the administration of the anlotinib plus platinum‐etoposide as the first‐line treatment for ES‐SCLC patients, and achieved both benefits of PFS and ORR, as well as the patients in the subgroups of liver metastases and brain metastases. The study had a favorable safety profile with a 40% incidence of grade 3–4 AEs and no grade 5 AEs. No pneumonia or pulmonary AEs were recorded as well.

A total of 36 patients were enrolled from August 10, 2018 to January 16, 2020, and 1 withdrew the Inform Consent before medication. Thirty‐five patients were included in the efficacy and safety analysis finally. Data were cutoff on December 2, 2020. After six cycle treatments of the proposed regimen, the median PFS, OS, and ORR were 8.02 months (95% CI: 6.90 m, 9.66 m), 15.87 months (95% CI: 10.38 m, 18.89 m), and 85.71% (95% CI: 69.74%95.19%), respectively. These results were more significant compared to chemotherapy alone (PFS, 5.7 months; OS, 8.9 months; ORR, 55.3%), and bevacizumab + chemotherapy^17^ (PFS, 6.7 months; OS, 9.8 months; ORR, 58.4%). As of December 2, 2020, there were 23 OS events occurred with an OS maturity of 65.71%, which suggested a more survival clinical benefit compared with ICI + chemotherapy.^18^

In addition, according to the 2021 NCCN guidelines for SCLC, patients with relapse time >6 months after undergoing the first‐line therapy for ES‐SCLC could individually select the original therapeutic regimen.^19^, ^20^ In this study, anlotinib + EP followed by anlotinib maintenance treatment, with 31 patients with PD: 15 entered post‐line therapy (11 received chemotherapy, two received chemotherapy + ICI, two received radiotherapy), and the overall percentage of those who had received or had access to second‐line therapy was 70.97% (data not show), also further suggesting that patients could have more chances to reselect back to first‐line chemotherapy in the second‐line period to improve their overall survival.

In the real world, limited patients have access to second‐line treatment, and low PS scores after first‐line treatment become a reason to stop receiving post‐line treatment.^21^ The proposed regimen in the present study included chemotherapy + anlotinib for 4–6 cycles, followed by maintenance treatment with anlotinib, in which a median PFS of 8.02 months could be achieved, thereby enabling patients to enter a second‐line therapy, eventually resulting in the extension of OS.

The follow‐up period of this study was every two cycles (42 days) over 12 cycles (8 months) and every three cycles (63 days) starting from cycle 13. This ensures timely detection of disease progression and increases the accuracy of determining PFS.

Brain metastasis is associated with a poor SCLC prognosis. Approximately 10%–25.8% of patients with SCLC at diagnosis had symptomatic or asymptomatic brain metastases. First‐line immunotherapy studies that enrolled ES‐SCLC patients with asymptomatic or stable brain metastases for treatment included the IMpower133,^6^ CASPIAN, and KEYNOTE‐604 trials.^22^ Nonetheless, only in the CASPIAN^23^ study (durvalumab plus chemotherapy) was the OS of all patients improved (with or without brain metastasis at baseline) compared to chemotherapy alone.

The multicenter, randomized, double‐blind, phase II ALTER1202 trial assessed anlotinib in patients with limited‐stage SCLC or ES‐SCLC who had developed progression after two lines of chemotherapy. In a subgroup of third‐line and post‐line extensive‐stage SCLC combined with stable brain metastases, anlotinib alone (*n* = 21) versus placebo (*n* = 9) had a PFS of 3.8 m versus 0.8 m (HR = 0.15) and a DCR of 71.4% versus 11.1% (*P* = 0.004).^24^ It indicates that anlotinib has a control effect on stable brain metastases in SCLC. And in this study, patients who were allowed into the combination and had stable brain metastases, as determined by imaging data of brain lesions alone, this subgroup had a PFS of 7.34 m (95% CI: 5.68 m, 9.20 m), OS of 10.58 m (95% CI: 7.33, NE), ORR of 87.5% (95% CI: 47.35, 99.68), DCR was 100% (95% CI: 63.06, 100.00). It demonstrated that both anlotinib + EP provided a good survival benefit in patients with stable recurrence‐free brain metastases.

The CASPIAN, IMPOWER‐133, KEYNOTE‐604, Reck‐2016, and ECOG‐ACRIN‐5161 studies included a subset of people with combined liver metastases and explored the benefit of ICI + EP in patients with liver metastases from ES‐SCLC: the HR of OS was 0.84.^25^ liver metastases subgroup in the ALTER1202 showed the HR of OS was 0.54 for anlotinib alone (n = 27) versus placebo (*n* = 12). In contrast, OS in IMpower133 was 9.3 m. However, as determined by imaging data of liver lesions in patients with liver metastases from this regimen alone in the present study, the subgroup with liver metastases had a PFS of 7.33 m (95% CI: 4.76 m, 9.69 m), OS of 10.38 m (95% CI: 5.22, 15.87), ORR of 76.92% (95% CI: 46.19, 94.96), and DCR of 84.62% (95% CI: 54.55, 98.08). In conclusion, anlotinib + EP can have a trend of benefit in the population with liver metastasis ES‐SCLC.

The reason for the aforementioned results might be that anti‐vascular‐targeted drugs combined with platinum‐etoposide improved the survival of patients with ES‐SCLC, and the safety was controllable. It also had a good performance in liver and brain metastasis. This encouraging result might be due to the favorable transmembrane effect of anlotinib hydrochloride and its synergistic effect with chemotherapy.

Grade 3 to 4 AEs commonly observed in this study were hand–foot syndrome (17%) and hypertriglyceridemia (11%), which are also the common adverse responses of anlotinib monotherapy, similar with other study.^14^ In addition, it was found that the incidence of hand‐foot syndrome was positively correlated with the effectiveness of anlotinib.^26^ Meanwhile, hypertension and hand‐foot syndrome were noted as independent protective factors of PFS in patients with NSCLC underwent anlotinib treatment.^27^

The present study was a greater safety compared to Atezolizumab + EP, and Durvalumab + EP ± Tremelimumab.^6^, ^23^ No patient developed pneumonia or pulmonary associated AEs, making the therapy in this study significantly different from ICI + chemotherapy, which the incidence of grade 3–4 pneumonia was 2%.^10^

In this study, among the 35 subjects, a total number of three grade 3 AEs that led to discontinuation (two of nausea and vomiting, one of malaise once), none of the above grade 3 AEs were severe and difficult to salvage (e.g., proteinuria). The shorter duration of chemotherapy with our regimen (compared to other first‐line treatments) has the potential to improve patient quality of life and adherence. Overall, anlotinib showed clinical benefits and a tolerable safety in a considerable number of patients with ES‐SCLC.

This study had some limitations. This was a single‐arm study with small sample size. And the PFS and ORR were not achieved to the preset 9 months and 80%. However, PFS in this study was 8.02 months with the lower 95%CI limit was 6.09 months, which was higher than the historical control of 5.2 months. While ORR was 85.71% with the lower 95%CI limit was 69.74%, which also higher than the historical control of 55%. Therefore, this study met the primary endpoint. Studies with a large sample size are further needed to validate these findings. Meanwhile, the comparison with chemotherapy alone was missing in this single‐arm study. However, the trial was designed based on historical data from clinical trials of chemotherapy alone, and statistical calculations also had been performed in combination with expected survival goals, including the sample size was estimated and compared with historical data. In addition, the biomarker with predictive value was hard to explore in the present study because of the small sample size. Although the ORR was very high, and the DCR was very close to 100%, it is still expected to be verified in a larger sample study. Actually, a multicenter randomized controlled trial is undergoing, which will provide more evidence on the efficacy and safety of chemotherapy alone and chemotherapy + anlotinib in ES‐SCLC patients.

In summary, this single‐arm phase II trial in the first‐line treatment of ES‐SCLC showed that the addition of anlotinib to platinum‐etoposide was associated with significantly improved PFS and ORR, accompanying with a safety profile.

The anlotinib + etoposide + platinum therapy showed promising prognosis (especially ORR and OS) and favorable tolerability profile in the first‐line treatment of ES‐SCLC, which needs to be demonstrated in studies of larger sample size.

## CONFLICT OF INTEREST

This study received funding from Chia Tai Tianqing Pharmaceutical Group Co., Ltd. The funder was not involved in the study design, collection, analysis, interpretation of data, the writing of this article or the decision to submit it for publication. All authors declare no other competing interests.

## AUTHOR CONTRIBUTIONS

CPH, HPY, and PBD contributed to the conception of the study; PBD, HPY, LMC, QHG, JA, LQ, ML, and BMH performed the experiment; PBD, CC, and JJ contributed significantly to data analysis and wrote the manuscript.

## ETHICS APPROVAL STATEMENT

The study was conducted in accordance with the International Conference on Harmonization Good Clinical Practice Guideline, the Declaration of Helsinki, and applicable local regulations with approval from an independent ethics committee or institutional review boards.

## PATIENT CONSENT STATEMENT

All patients provided written informed consent for participation.

## CLINICAL TRIAL REGISTRATION

Clinical Trials. Gov number, NCT04675697. https://clinicaltrials.gov/.

## Supporting information


Appendix S1

## Data Availability

The data that support the findings of this study are available from the corresponding author upon reasonable request.
