# Supplementary material for: Anlotinib plus platinum‐etoposide as a first‐line treatment for extensive‐stage small cell lung cancer: A single‐arm trial
Source: Cancer Med. 2022 May 8;11(19):3563–71. doi: 10.1002/cam4.4736 (PMC9554443; doi:10.1002/cam4.4736)
Supplement: Supplementary file 1 — Appendix S1 [file CAM4-11-3563-s001.docx]

# Supplement Table 1 The inclusion and exclusion criteria

| Inclusion criteria |
| --- |
| Patients included in the study met all the following criteria:   1. Aged 18–70 years 2. Rated as grade 0–2 in the ECOG whole-body status (PS), or grade 3–4 if induced by SCLC 3. With an expected survival no less than 3 months 4. With extensive-stage SCLC diagnosed pathologically (according to the staging standard introduced by the Veterans Administration Lung Study Group), and having a measurable lesion (a tumor lesion of ≥10 mm in long diameter in computed tomography (CT) scanning, or a lymph node lesion of ≥15 mm in short diameter in CT scanning, which had not received radiotherapy, cryotherapy, or other local therapies, according to the RECIST1.1 standard) 5. Having not received chemotherapy or immunotherapy 6. Some patients who received radiotherapy were included if the radiotherapy area was smaller than 25% of the bone marrow area; they did not undergo total pelvic or chest radiation; their previous radiotherapy ended at least 4 weeks before the inclusion; they had recovered from radiotherapy-induced acute toxicity reaction; and the local lesion that was irradiated was not included in the measurable lesion, unless significant progress was observed in the lesion after the last radiotherapy 7. Having normal major organ functions, that is, their organs met the following criteria: 8. Blood routine examination criteria: Absolute neutrophil count (ANC) ≥1.5 × 10^9^/L, blood platelet (PLT) ≥ 100 × 10^9^/L, and Hb ≥ 100 g/L (no blood transfusion or blood products in 14 days, and no Granu1ocyte colony-stimu1ating factor (G-CSF) or other hematopoietic stimulant corrections) 9. Biochemical examination criteria: Total bilirubin (TBIL) < 1.5 × ULN, ALT, AST, and Alkaline phosphatase (ALP) < 2.5 × ULN, BUN and Cr ≤ 1 × ULN, or endogenous creatinine clearance rate ≥50 mL/min 10. Females of childbearing age who took reliable contraceptives or had a negative pregnancy test (serum or urine) result 7 days before inclusion, and were willing to take appropriate contraception measures during the study and in 8 weeks after the last administration of the treatment drug; males who agreed to take appropriate contraception measures during the study and in 8 weeks after the last administration of the treatment drug or had undergone sterilization operation   Patients who voluntarily participated in the study, signed the informed consent, and were well compliant and cooperative in follow-up visits. |
| Exclusion criteria |
| Patients meeting any of the following were not included in the study:   1. With mixed small-cell carcinoma and non-small-cell carcinoma 2. With active CNS metastases and/or cancerous meningitis or found to have active CNS metastases and/or cancerous meningitis in examinations during the screening stage [Patients were included in the study if they: (1) had asymptomatic brain metastases (without progressive CNS symptoms induced by brain metastases, required no corticosteroids, and had a lesion size ≤1.5 cm), provided they underwent regular brain imaging examinations for the diseased site; (2) were treated and in a stable state, had no imaging evidence for new or enlarged brain metastases at least 2 weeks after brain metastasis treatment, and had discontinued steroids or anticonvulsants at least 14 days before the therapy started.] 3. With imaging findings showing invaded central great vessels or obvious pulmonary cavity or necrotizing tumor 4. With hypertension who were taking two or more antihypertensive drugs 5. With cardiovascular diseases, such as myocardial ischemia or myocardial infarction of grade II or above, uncontrolled arrhythmias, new functions of grade III to IV, or cardiac ejection fraction <50% 6. With abnormal coagulation function [international normalized ratio (INR) > 1.5 or prothrombin time > upper limit of normal value (ULN) + 4 s, or activated partial thromboplastin time (APTT) > 1.5 ULN], prone to bleeding, or receiving thrombolytic or anticoagulant therapy 7. Having significant blood in the cough or daily hemoptysis of 2.5 mL or more in 2 months before the inclusion 8. Having bleeding symptoms or definite bleeding tendency of significant clinical significance, such as gastrointestinal bleeding, hemorrhagic gastric ulcer, fecal occult blood ++ or above at baseline, or vasculitis, in 3 months before the inclusion 9. Having developed artery/venous thrombosis in 12 months before the inclusion 10. With known hereditary or acquired bleeding and thrombosis tendency 11. Having a wound or fracture that could not be healed for a long time 12. Having underwent major surgery or had severe traumatic injury, fracture, or ulcer in 4 weeks before the inclusion 13. Subjected to factors that significantly affected the absorption of oral medication 14. Having developed abdominal fistula, gastrointestinal perforation, or abdominal abscess in 6 months before the inclusion 15. Having the urine routine result suggestive of urine protein ≥++, or the 24-h urine protein amount confirmed as ≥1.0 g 16. With serous membrane effusion showing clinical symptoms and requiring symptomatic treatment 17. With active infections that required antimicrobial treatment 18. Having a history of psychotropic drug abuse and unable to quit or with a mental disorder 19. Having participated in other clinical trials on anti-tumor drugs 4 weeks before the inclusion 20. Previously or currently having other incurable malignancies 21. Having received over-potent CYP3A4 inhibitor treatment in 7 days before the inclusion, or having received over-potent CYP3A4 inducer treatment in 12 days before the inclusion 22. Pregnant or lactating women who were fertile but were unwilling or unable to take effective contraception measures   With other conditions that might clinically affect the conduct or outcome of the study as determined by researchers |
